# Biomineralizations: insights and prospects from crustaceans

**DOI:** 10.3897/zookeys.176.2318

**Published:** 2012-03-20

**Authors:** Gilles Luquet

**Affiliations:** 1 Biogéosciences, UMR 5561 CNRS - Université de Bourgogne, Dijon, France

**Keywords:** ACC, amorphous calcium carbonate, biomineralization, calcification, calcium storage, cuticle, organic matrix

## Abstract

For growing, crustaceans have to molt cyclically because of the presence of a rigid exoskeleton. Most of the crustaceans harden their cuticle not only by sclerotization, like all the arthropods, but also by calcification. All the physiology of crustaceans, including the calcification process, is then linked to molting cycles. This means for these animals to find regularly a source of calcium ions quickly available just after ecdysis. The sources of calcium used are diverse, ranging from the environment where the animals live to endogenous calcium deposits cyclically elaborated by some of them. As a result, crustaceans are submitted to an important and energetically demanding calcium turnover throughout their life. The mineralization process occurs by precipitation of calcium carbonate within an organic matrix network of chitin-proteins fibers. Both crystalline and stabilized amorphous polymorphs of calcium carbonate are found in crustacean biominerals. Furthermore, Crustacea is the only phylum of animals able to elaborate and resorb periodically calcified structures. Notably for these two previous reasons, crustaceans are more and more extensively studied and considered as models of choice in the biomineralization research area.

## Introduction

Biomineralization corresponds to the process of mineralized structures formation by living organisms. This word designates also the elaborated mineralized structure itself. This phenomenon, which appeared firstly in Eubacteria and Archea as a biologically-induced process (Lowenstam 1981), is widespread in the Metazoa kingdom as a biologically-controlled process mediated by an organic matrix (Mann 1983), also termed template-directed mineralization (Pouget et al. 2009). The first major function of this process is the hardening of a skeleton, a structure that provides support for muscles and protection against environmental pressures (Lowenstam and Weiner 1989, Simkiss and Wilbur 1989). The fossil species, discovered so far, revealed that the first calcified metazoan exoskeletons appeared probably at the end of the Precambrian period, during the Proterozoic (Kirschvink and Hagadorn 2000, Knoll 2004). The oldest known crustacean is dating 520 million years, discovered in the Early Cambrian Maotianshan Shale Lagerstätte, in China (Chen et al. 2001). The reasons for the appearance and selection of the biomineralization process during evolution remain speculative but several hypotheses have been mentioned including protection against predators. The adverse evolution of the ionic composition of the primitive ocean, notably the calcium ion concentration, was also raised.

The biomineralization process is well developed in several taxa, including Crustacea as one of the most outstanding group in this respect. As a consequence of the presence of a rigid exoskeleton, the growth and the whole physiology of these animals are linked to molting cycles characterized by the complete renewal of the exoskeleton during each molting. All of the arthropods harden their new cuticle by a process called sclerotization (protein-polysaccharide and protein-protein cross-linking by the way of quinonoid-sclerotizing agents) and, in addition, most of the crustaceans proceed furthermore by calcification.

The major source of calcium used for exoskeleton calcification is exogenous: the water in which most crustaceans live. In seawater, calcium concentration is very high but many species also live in freshwater or on land, where the availability of calcium at ecdysis may be low or even absent. Therefore crustaceans have developed different strategies to solve the problem of calcification, in particular by storing calcium during the premolt period (Graf 1978, Greenaway 1985), a phenomenon especially well developed in terrestrial species of amphipods, isopods and decapods. The odd thing is that calcium storage is also found in some aquatic species. The food (including sometimes the exuviae), a possible source of calcium ions, represents a minor contribution to the cuticle calcification, whatever the way of life of the animal considered.

Next, crustaceans are particularly interesting because of their active calcium metabolism and their ability to form and resorb cyclically (more or less partially) not only an external calcified structure but also, in many species, calcium storage biomineralizations.

Another characteristic of calcium metabolism in crustaceans are the calcium-transporting epithelia very similar to some vertebrate ones, and in this way they represent also good models to understand how they function. Calcium pumps and enzymatic systems, similar to those associated with vertebrate calcium-transporting epithelia, have been evidenced in the cuticular epidermis and the calcium storage epithelium. Furthermore crustaceans are convenient models for studying the hormonal regulation of the calcium turnover with the possible involvement of vertebrate-type hormones such as calcitonin/CGRP and vitamin D presumably evolving from invertebrate counterparts (McWhinnie et al. 1969, Greenaway 1985, Fingerman et al. 1993, Luquet and Marin 2004).

## Molting cycle, cuticle and calcification

In crustaceans, a cellular hypodermis, which underlies the calcified cuticle also called carapace, is responsible for the complete synthesis of the exoskeleton. This cuticle comprises four main layers, from the external to the innermost layer: the epicuticle, the exocuticle, the endocuticle and the membranous layer. Calcification of this cuticle has been particularly well studied in Decapoda (Travis 1963, Roer 1980, Greenaway 1985). Except for the arthrodial cuticle present at the joints of appendages and at the base of gills and setae, for the cuticle covering the gills and the gut, and for the membranous layer of the exoskeleton, the three other cuticlar layers are more or less mineralized. The process occurs essentially by precipitation of calcium carbonate into an organic matrix network of chitin-protein fibers arranged in a twisted plywood and honeycomb-like structure (Bouligand 1972, Giraud-Guille et al. 2004).Calcification of the carapace takes place atdifferent sites within the cuticle: around chitin-protein fibers, at the level of interprismatic septa, around and within pore-canals formed by cytoplasmic extensions of hypodermal cells (Roer and Dillaman 1984, Giraud-Guille 1984a, 1984b, Compère et al. 1992, 1993, Giraud-Guille et al. 2004, Romano et al. 2007). Until recently it was thought that the calcification of the decapod cuticle occurred mainly in a crystalline form (calcite or Mg-calcite). Some recent investigations confirmed the presence of a crystalline polymorph but revealed that ACC and ACP are also present in various amounts, depending on the species concerned (Dillaman et al. 2005, Romano et al. 2007). For example, by following the early hours of the carapace calcification of the blue crab, *Callinectes sapidus*, Dillaman and co-workers (2005) evidenced that calcium carbonate is first deposited as ACC, which then transforms into calcite. Other considerations came from the extensive study of the American lobster, *Homarus americanus*, cuticle (Sachs et al. 2006, Romano et al. 2007, Raabe et al. 2008, Al-Sawalmih et al. 2008, 2009, Fabritius et al. 2009, Nikolov et al. 2010, 2011). They defined 7 hierarchical levels of cuticle organization, from the polymerization of N-acetyl-glucosamine (level I) to the finished product, the complete calcified cuticle (level VII). Level IV corresponds to the deposition of calcium carbonate around chitin-protein nanofibers. On the other hand, they demonstrate that only the outer part of the exocuticle of the American lobster is calcified with calcite/Mg-calcite, whereas the rest of the exocuticle as well as the endocuticle are completely calcified with ACC and, in a lesser extent, with ACP. They also analyzed the relations referring to the multiple levels of the cuticle complex structure, from the nanoscale to the macroscopic level, and its remarkable mechanical properties as an inspiration source for biomimetic materials research.

Until recently, the structure and calcification of the cuticle in isopods have been poorly investigated (Price and Holdich 1980a, 1980b, Wood and Russell 1987, Štrus and Compère 1996). Recent works have shown that the different layers of isopod cuticle could be more or less and irregularly calcified and that the composition and distribution of the mineral could vary considerably from the decapods (Becker et al. 2005, Hild et al. 2008, 2009, Seidl et al. 2011, Neues et al. 2007, 2011). If amorphous calcium carbonate has been demonstrated to be transitorily present as precursor of a crystalline polymorph (calcite essentially), similarly to decapods, stabilized ACC and in a lesser extent ACP have been evidenced as components of isopod cuticle. For example, in *Armadillidium vulgare* and *Porcellio scaber* (Neues et al. 2007, Hild et al. 2008), the epicuticle and the membranous layers are not mineralized, the exocuticle contains both Mg-calcite and ACC/ACP whereas the endocuticle is only calcified with ACC. The thickness of the endocuticle appears correlated with the behavior of these terrestrial crustaceans for avoiding a possible environmental danger: *P. scaber* avoids predation by running away, which requires a thin and flexible cuticle (with 16% Mg-calcite, 38% ACC and 12% ACP), whereas *A. vulgare* avoids predation by rolling into a sphere and thus possesses a thicker and more mineralized cuticle (with 12% Mg-calcite, 59% ACC and 11% ACP). Nevertheless a comparative study performed on 4 marine and 6 terrestrial isopod species revealed a great variation of the composition, more pronounced in the marine species even with similar habitats and behaviors: 12–20% Mg-Calcite, 38–59% ACC and 0–14% ACP for the terrestrial species *versus* 4–58% Mg-calcite, 17–60% ACC and only 0–3% ACP for the marine species.

Finally, the cuticle of *Ligia italica* was used as a model of matrix-mediated calcification in a comparative study using also calcite deposits generated by *Synechoccocus* cyanobacteria as a biologically-induced calcification model. As previously shown for growth zones of bones (Carlisle 1970, Landis et al. 1986), the authors demonstrate that silicon, in the form of amorphous oligomerized silicic acid, is involved in the early steps of calcification at nucleation sites, serving as intermediary between polysaccharide-protein complexes and inorganic ions catalyzing the precipitation of a mineral phase (Matsko et al. 2011).

While the presence of ACC as the main polymorph in calcium storage deposits can be easily understood (see below), the presence of metastable ACC at the level of the cuticle is more surprising. Nevertheless, some advantages of the presence of stable amorphous minerals in cuticles are shape flexibility, plasticity, as well as optimization of strenght and toughness (Al-Sawalmih et al. 2009). Another interesting feature of ACC is that it enables easier calcium mobilization, useful for the partial decalcification of the old cuticle in every premolt as well as for cuticle repairing in intermolt. On the other hand, it has been suggested for isopods that the structure and mineralization of the cuticle can be related to the ecophysiology of the animals considered.

From the structural-mineral point of view, the presence of different polymorphs of calcium carbonate within the crustacean cuticles is tightly linked to the presence of specific matrix molecules.

Some molecules, identified as cuticular proteins, have been characterized, the precise function of which is not well elucidated. Some of them, possessing a Rebers-Riddiford domain in their sequence, a hallmark of their chitin-binding ability (Rebers and Willis 2001), are probably involved in the formation of the organic network serving as template for the precipitation of calcium carbonate. An 18-residue domain, also called cuticle_1 domain, has been found in proteins belonging exclusively to hard cuticle. They are suspected to play a role in the regulation of calcite crystal growth (Kragh et al. 1997, Nousiainen et al. 1998, Andersen 1999).

Other proteins, with *in vitro* calcium-binding ability or which interact with CaCO_3_ formation, are also thought to play an active role in the *in vivo* CaCO_3_ precipitation process. Among them are DD4/crustocalcin from *Penaeus monodon* (Endo et al. 2000, 2004), CAP-1 and CAP-2 from the crayfish *Procambarus clarkii* (Inoue et al. 2001, 2003, 2004, 2007, Sugawara et al. 2006,Yamamoto et al. 2008) and more recently Casp-2 from the blue crab *Callinectes sapidus* (Inoue et al. 2008) but their real *in vivo* functions remain speculative. Other technical approaches have been used recently allowing the simultaneous characterization of multiple transcripts encoding putative cuticular proteins. First, an EST database was produced from two cDNA libraries prepared from the gill and the hypodermis of the blue crab, *Callinectes sapidus* (Coblentz et al. 2006). By using 3 different strategies for screening this database, 73 transcripts were suggested to code for cuticular proteins (Faircloth and Shafer 2007). Efforts are currently made for obtaining the complete sequence of these transcripts and for determining if they encode calcified cuticle proteins versus arthrodial membrane proteins (Wynn and Shafer 2005, Shafer et al. 2006, Faircloth and Shafer 2007). The second approach aimed to develop a cDNA microarray chip for *Portunus pelagicus* for generating expression profiles of genes involved in the cuticle formation. Twenty-one differentially expressed transcripts (up-regulated in postmolt) encoding cuticular proteins were isolated (Kuballa et al. 2007). Blast analyses were performed against cuticleDB, a database comprising all the proteins of Arthropod cuticle identified so far (Magkrioti et al. 2004). The comparison was made on the base of the presence of specific domains. Thirteen of these 21 transcripts contain the cuticle_1 domain specific for hard cuticle, 4 contain a variant of the RR domain (chitin_bindin_4) found in both calcified and uncalcified cuticle and 4 possess a domain called Pfam B 109992 found associated to a cuticular protein, CPCP1876, from the rock crab *Cancer pagurus*. In another study using the same approach, Kuballa and Elizur (2008) focused on transcripts possibly associated with mineralization and sclerotization of cuticle organic matrix. More particularly, they suggested that, because of their affinity for glycoproteins, C-type lectin receptors and a mannose-binding protein (MBP1) could be involved in the regulation of calcification by two alternative pathways involving glycosylation and deglycosylation events of cuticle proteins linked to conformational changes. They are also thought to play a role in the activation of the phenoloxidase pathway. Finally, by generating two cDNAs libraries, one from the whole body, the other from specific organs (brain, eystalks, mandibular organ, Y-organs) 556 clones were sequenced among which 14% encoding cuticular proteins (Kuballa et al. 2011). According to the cuticleDB nomenclature (Magkrioti et al. 2004) cuticular proteins up-regulated in postmolt were identified: CUT proteins (CUT1 to CUT8, CUT12 and CUT13), the DB1, DB2 and DB3 proteins, the CB3 and CB4 proteins, the VR2 and VR3-like proteins, the DBM protein. More curiously, a transcript encoding the gastrolith GAP65 protein was also found suggesting first that this protein is not specific for the gastrolith disc and second that this protein is probably involved in the calcification of the cuticle, based on its role in gastrolith formation (Shechter et al. 2008; see also below the paragraph untitled “Calcium storage, In Decapoda”).

## Calcium storage

Except for the Copepoda and Cirripedia (Maxillopoda), calcium storage is a process commonly found in the other groups of crustaceans. The sites of storage as well as the morphologies of the storage structures are very diversified (Graf 1978, Luquet and Marin 2004). Nevertheless, it seems that a general feature is the storage of calcium carbonate in an amorphous polymorph. The stored calcium must be quickly available after ecdysis and the amorphous polymorph of calcium carbonate (ACC) is the most compatible with this function. Chemical inorganic ACC is a metastable polymorph, which transforms immediately into a crystalline polymorph, calcite being the most stable. Even though biogenic ACC is stabilized in time by matrix molecules, it remains easily mobilizable (Addadi et al. 2003).

### In Amphipoda (Malacostraca: Peracarida)

The most extensively studied model is the semiterrestrial talitrid amphipod *Orchestia cavimana*. This animal cyclically stores calcium in two diverticula of the midgut, called posterior ceca (PC), in the form of calcareous concretions (Figure 2; Graf and Meyran 1983, 1985, Meyran et al. 1984, 1986).

**Figure 1.**
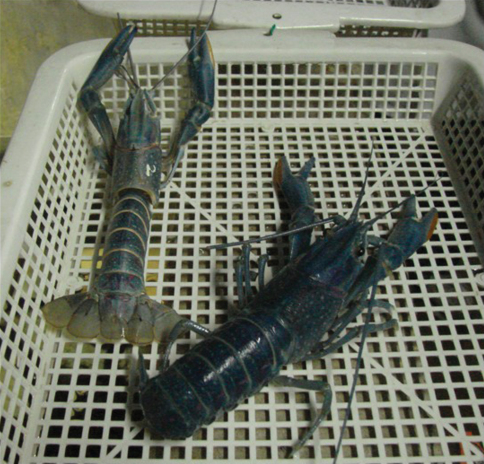
Adult specimen of the freshwater red claw crayfish, *Cherax quadricarinatus*, just after molting (at left the exuviae).

**Figure 2.**
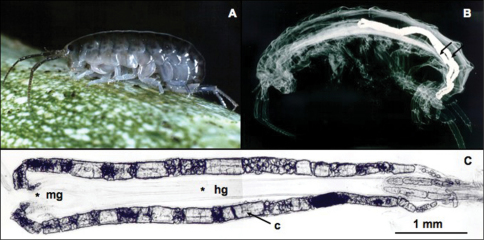
Calcium storage in the semiterrestrial amphipod, *Orchestia cavimana*. **A** Adult male specimen (2-cm long) **B** Radiography of an adult specimen just after ecdysis (electron-dense stored calcium is well visible in 2 diverticula of the midgut; arrows) **C** Calcium is stored as calcareous concretions in paired posterior ceca. c: concretion, hg: hindgut, mg: midgut.

The storage organs are composed of a one-layered epithelium forming tubules, which are proximally connected to the midgut and distally blind-ended. During the premolt period, calcium originating from the present cuticle is transported in an ionized form and is precipitated in the PC lumen within an organic matrix synthesized by the PC cells (Figure 2C). Concretions are formed by addition of successive concentric layers of organic matrix, within which calcium carbonate is precipitated as the amorphous polymorph (Raz et al. 2002). After ecdysis, calcium resorption occurs by successive generations of 1 µm diameter calcified spherules that form at the apical part of the PC epithelium and dissolve at the basal part of the extracellular PC network. The storage proceeds exponentially during a 16-day mean period for an adult specimen concomitant with the partial demineralization of the cuticle, whereas dissolution of concretions is performed in less than 48 h. The stored calcium represents 60% of the calcium necessary for the complete calcification of each new brand cuticle.

To understand the formation of biomineralized structures, the characterization of the molecular components of the organic matrix is of first interest. Biochemical and molecular biology techniques were used to characterize proteinaceous components of the matrix. One peculiar protein, named Orchestin, has been well characterized and completely sequenced. This phosphorylated calcium-binding protein is probably responsible for the precipitation of calcium carbonate within the storage organs in premolt as well as for the formation of the calcium resorption spherules in postmolt (Testenière et al. 2002, Hecker et al. 2003, 2004). Orchestin is also thought to be involved in the determination of the amorphous calcium carbonate (ACC) polymorph, in cooperation with other matrix molecules (Hecker et al. 2004).

Other interesting amphipod models are troglobites of the genus *Niphargus*. They are able to store calcium in posterior ceca as amorphous calcium carbonate concretions, similarly to *Orchestia* amphipods, but also as rhomboedric crystalline structures (probably calcitic) within the gut (Graf 1975).

### In Isopoda (Malacostraca: Peracarida)

Isopods possess a particular biphasic mode of molting: they shed first the posterior half of their cuticle, then the anterior part (Messner 1965, Steel 1993, Ziegler 1994). During the premolt period, oniscid isopods elaborate calcified deposits in the four anterior sternites between the cuticle and the hypodermis. The formation of such sternal plates has been particularly well studied in the woodlouse, *Porcellio scaber* (Ziegler 1994, 1997, Fabritius and Ziegler 2003, Ziegler et al. 2005). Some results were also obtained from isopods of the genus *Oniscus* (Steel 1993), *Ligidium* (Ziegler and Miller 1997, Glötzner and Ziegler 2000) and *Ligia* (Numanoi 1934, Ziegler and Miller 1997, Glötzner and Ziegler 2000, Štrus and Blejec 2001, Ziegler et al. 2007).

The calcified storage structures are composed of amorphous hydrated calcium carbonate precipitated within an organic matrix of spherules (Ziegler 1994, Ziegler and Miller 1997, Becker et al. 2003, Ziegler et al. 2005).

The formation and resorption of these sternal plates seem closely related to the biphasic molting cycle of these crustaceans. The calcium deposits, fully developed before ecdysis of the posterior part, are completely resorbed before ecdysis of the anterior part. It has been suggested that the calcium, stored as calcospherules in the edysial space, is used to calcify the posterior cuticle (Steel 1993, Štrus and Compère 1996, Ziegler and Merz 1999, Ziegler and Scholz 1997). After ecdysis of the anterior cuticle, the stored calcium is resorbed by the epidermis and transported until the cuticle through the haemolymph in a ionic form (Ziegler and Scholz 1997, Ziegler et al. 2005, 2007).

### In Decapoda (Malacostraca: Eucarida)

The order Decapoda represents the largest group of crustaceans living on land as well as in water, and storage strategies are well developed and very diversified in decapods.

For calcifying the cuticle, calcium ions are translocated from an endogenous or exogenous source through the haemolymph. In some species this medium is used as a storage site. In the freshwater/land crab, *Holthuisana transversa*, the haemolymph has been observed to contain small-size calcified granules representing a way of transporting a great amount of calcium in a short period while avoiding toxification (Sparkes and Greenaway 1984).

Hepatopancreas is also used by some crabs like *Cancer pagurus*, *Carcinus maenas*, *Callinectes sapidus* and *Paratelphusa hydrodomous* as a storage organ where calcified granules of calcium phosphate have been found, also considered by some authors as playing a role in metal detoxication processes (Adiyodi 1969, Becker et al. 1974, Guary and Negrel 1981). This was also evidenced more recently in the land crab *Ucides cordatus* (Corrêa et al. 2002, 2009).

**Figure 3.**
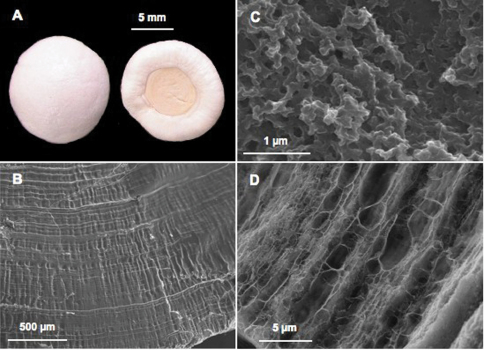
Calcium storage as gastroliths in decapods. **A** Pair of gastroliths from the crayfish *Cherax quadricarinatus* (Light Microscopy) **B** Internal striated structure visible on natural fracture after slight acetic acid decalcification (SEM) **C** Ultrastructure well visible after natural fracture and high magnification (SEM): the mineral is precipitated as nanospheres **D** Organic matrix network revealed after acetic acid decalcification (SEM).

Finally some decapods store calcium in their cardiac stomach wall between the one-layered epithelium and a cuticle as so-called gastroliths (Travis 1960, 1963). They appear as paired semi-spherical structures in lobsters and crayfishes (Figure 3A) and as four more irregular deposits in gecarcinid land crabs. After acidic decalcification, we observed an important network of concentric and transversal micro- and nanofibers of organic matrix forming meshes of different sizes (Figure 3B and C) within which calcium carbonate is precipitated as nanospheres, as generally found in all the ACC biomineralized structures (Figure 3B).

Four organic matrix proteins from gastroliths have been well characterized and sequenced so far. The first one, named GAMP, was obtained from the crayfish *Procambarus clarkii* (Ishii et al. 1996, 1998). More recently GAP65, GAP10 and crustacyanin-A2 subunit were obtained from the Australian red claw crayfish, *Cherax quadricarinatus* (Shechter et al. 2008, Luquet et al. 2009, Glazer et al. 2010). Among these proteins, only GAP65 has been suggested to be involved in the determination and stabilization of the amorphous polymorph.

How the biogenic ACC polymorph can be stabilized in time is still an open question that received recent attention. It was previously suggested that specialized macromolecules (acidic proteins, phosphoproteins, sulfated glycoproteins) or ions such as magnesium or phosphate could contribute to this stabilization (Aizenberg et al. 1996, 2002, Raz et al. 2000, 2003, Addadi et al. 2003, Luquet and Marin 2004, Marin and Luquet 2007, Shechter et al. 2008, Bentov et al. 2010). Very recently, Sato and co-workers (2011) and Akiva-Tal and co-workers (2011), by using cucticle and/or gastrolith from *Procambarus clarkii* and *Cherax quadricarinatus* as models, respectively, presented a new insight into induction and stabilization of ACC. By using solid state NMR spectroscopy, they demonstrated the presence of phosphorylated energy-rich intermediates of the glycolytic pathway and suggested their possible involvement in these processes. ACC particles would form first due to the interaction of specialized matrix macromolecules bound to chitin in the CaCO_3_ precipitation process. Then phosphoenolpyruvate (PEP) and 3-phosphoglycerate (3PI) would act by binding to the surface of ACC, thus inhibiting the transformation of ACC into a crystalline polymorph (Sato et al. 2011). Citrate might be also involved in ACC stabilization by forming citrate-Ca^2+^-P complexes (Akiva-Tal et al. 2011). Similarly, it is to notice that NMR analyses revealed that the amorphous mineral storage structures found in the hepatopancreas of the crab, *Ucides cordatus*, are phosphate-rich granules containing mainly orthophosphate, but also pyrophosphate and glucose-6-phosphate, considered as possibly involved in the stabilization of the amorphous state (Corrêa et al. 2009).

## Conclusion

The formation of the majority of biominerals is under biological control. The knowledge of the physical and chemical features of the matrix components (proteins, polysaccharides, proteoglycans, lipids, low-molecular weight components...) is a prerequisite to understand, at the molecular level, how a biomineralization is elaborated, how the matrix molecules are involved in the nucleation and precipitation processes, how they influence the determinism of the polymorph obtained, and finally how a demineralizing process may occur.

In consideration of their ability to cyclically elaborate and resorb biomineral composites, crustaceans appear as convenient models for such prospects. They are not only able to synthesize a calcified exoskeleton but also calcium storage structures, which differ in their morphology and in their mineral composition. The calcium storage deposits are transient reservoirs of calcium ions that must be quickly mobilizable after ecdysis, and, for this reason, the storage structures are composed mainly of ACC. The cuticle reveals also a remarkable biocomposite because of the simultaneous presence of different polymorphs of CaCO_3_ and ACP as well. Recent investigations suggest that if some general structural features may be common to all cuticles, it seems likely that the cuticle mineral composition, linked to the molecular content, not only could vary from one order of crustaceans to another but could also depend on the ecophysiology of each species.

To understand why the amorphous calcium carbonate state is stabilized in time whereas the same purely inorganic mineral is completely unstable, as well as how the switch of the transformation from the amorphous to crystalline phases occurs, is also of great interest in the biomaterial and nanotechnology fields (Han and Aizenberg 2008, Gower 2008, Tao et al. 2009). From studies on crustaceans models it was evidenced that magnesium and phosphate ions, proteinaceous macromolecules and low-molecular weight phosphorylated components of the organic matrix could be responsible for this stabilization. If some recent results appear meaningful in this sense, unfortunately there are still too few matrix molecules really well characterized so far in crustaceans, as in other phyla, to have a clear idea of the complete process of elaboration of a biomineralization. Nevertheless, it seems more and more evident that the stabilization of the amorphous state, similarly to other processes such as CaCO_3_ nucleation and precipitation and the simultaneous presence of different polymorphs of CaCO_3_ are multi-parameter phenomena, which result from the synergistic cooperation of several if not all the categories of matrix components above described.

Finally, by means of comparative studies performed in other calcifying phyla, crustaceans are useful to determine why and how calcification could have emerged on Earth. The sequence analysis of the matrix proteins and the genes encoding these proteins could lead to the understanding of the strategy used by evolution to built and select different mineralizing systems: convergence of different biological systems for a similar mineralizing function by exaptation of initially non-mineralizing molecules or evolution and adaptative divergence from an ancestral biomineral system still undeciphered?
